# Electrospun Nanofiber Platforms for Advanced Sensors in Livestock-Derived Food Quality and Safety Monitoring: A Review

**DOI:** 10.3390/s25226947

**Published:** 2025-11-13

**Authors:** Karna Ramachandraiah, Elizabeth M. Martin, Alya Limayem

**Affiliations:** 1 Department of Biological Sciences, College of Arts & Sciences, University of North Florida, Jacksonville, FL 32224, USA; karna.r@unf.edu; 2 Food Science, Department of Biological and Agricultural Engineering, University of Arkansas, Fayetteville, AR 72701, USA; emartin@uark.edu

**Keywords:** electrospinning, nanofiber sensors, optical sensors, electrochemical detection, electronic noses, biosensors, food safety, real-time monitoring

## Abstract

Over the past two decades, the meat industry has faced increasing pressure to prevent foodborne outbreaks and reduce economic losses associated with delayed detection of spoilage. This demand has accelerated the development of on-site, real-time sensing tools capable of identifying early signs of contamination. Electrospun nanofiber (NF) platforms have emerged as particularly promising due to their large surface area, tunable porosity, and versatile chemistry, which make them ideal scaffolds for immobilizing enzymes, antibodies, or aptamers while preserving bioactivity under field conditions. These NFs have been integrated into optical, electrochemical, and resistive devices, each enhancing response time and sensitivity for key targets ranging from volatile organic compounds indicating early decay to specific bacterial markers and antibiotic residues. In practical applications, NF matrices enhance signal generation (SERS hotspots), facilitate analyte diffusion through three-dimensional networks, and stabilize delicate biorecognition elements for repeated use. This review summarizes major NF fabrication strategies, representative sensor designs for meat quality monitoring, and performance considerations relevant to industrial deployment, including reproducibility, shelf life, and regulatory compliance. The integration of such platforms with data networks and Internet of Things (IoT) nodes offers a path toward continuous, automated surveillance throughout processing and cold-chain logistics. By addressing current technical and regulatory challenges, NF-based biosensors have the potential to significantly reduce waste and safeguard public health through early detection of contamination before it escalates into costly recalls.

## 1. Introduction

In recent years, the meat industry has increasingly prioritized the development of sustainable and health-promoting products with extended shelf life to meet consumer expectations and minimize waste [1,2]. Despite these advances, significant post-production losses continue to challenge the sector. Estimates indicate that in the United States alone, approximately 38% of meat, 41% of poultry, and 33% of seafood are lost or discarded at the retail and consumer stages [2]. Similar patterns have been reported across Europe and industrialized regions of Asia, where most of the meat waste occurs downstream in the supply chain, particularly during retail handling and household consumption [2]. According to the latest OECD-FAO Agricultural Outlook (2025), about 13.5% of carcass meat is lost during slaughter and processing, and an additional 12.2% is wasted during distribution and consumption [3]. These losses emphasize the lack of real-time analytical tools capable of detecting spoilage. In the meat industry, spoilage refers to the deterioration of sensory and physicochemical quality due to microbial proliferation and oxidative degradation, leading to off-odors, discoloration, and the formation of volatile nitrogenous compounds such as ammonia and biogenic amines [1,3]. Moreover, these widespread inefficiencies indicate an urgent need for advanced monitoring tools capable of ensuring product quality and safety throughout storage and distribution.

Recent advances in materials science, mainly electrospinning, have yielded nanostructured sensing platforms with unprecedented control over surface area, porosity, and functionality, enabling sensitive and rapid detection of biochemical changes in foods [4]. Conventional analytical techniques such as high-performance liquid chromatography (HPLC), gas chromatography–mass spectrometry (GC–MS), and microbial plating remain gold standards for accuracy and precision; however, they are inherently incompatible with the dynamic and decentralized nature of modern food supply systems. These methods are time-intensive, often requiring several hours or days laboratory-bound, destructive to samples, and dependent on skilled personnel, rendering them impractical for real-time, in-field, or at-line monitoring [4,5]. This analytical gap has generated a critical need for sensor technologies capable of providing rapid, on-site, non-destructive, and cost-effective analyses. An effective food sensor must combine high sensitivity and specificity with affordability, portability, and user-friendliness, while permitting seamless integration into packaging materials or production lines [6,7,8]. The performance of any sensor is fundamentally determined by the physicochemical properties of its sensing interface or transducing element. An optimal sensing platform should therefore exhibit an extensive surface area for analyte interaction, efficient signal transduction, chemical and mechanical stability for supporting biological or chemical recognition elements, and rapid response kinetics [7,8]. Within this context, electrospun nanofibers (NFs) have evolved from a niche research material to a transformative platform in sensor design [9,10].

Electrospinning is a versatile and scalable fabrication method in which an applied high voltage pulls a polymer into continuous nanofibers [11,12]. These fibers accumulate into non-woven mats with structural and functional features that make them exceptionally attractive for sensing applications. Among the most important attributes of electrospun nanofibers is their high surface-to-volume ratio, which maximizes the active area available for analyte binding, catalyst immobilization, or interaction with external stimuli, thereby amplifying the sensor signal [13]. Their porous and interconnected architecture facilitates the rapid diffusion of gaseous or liquid analytes through the fiber network, resulting in shorter response times than those observed in dense, cast films [14]. Electrospun nanofibers (NFs) have also been utilized for charge and energy transfer, making the fibers strongly photoresponsive and suitable as sensing materials [15].

The fibers also offer versatile functionalization capabilities. Active compounds such as dyes, nanoparticles (NPs), or conductive polymers can be incorporated directly into the spinning solution (in situ functionalization) or introduced after fabrication via surface grafting, chemical reduction, or dip-coating (ex situ modification) [16,17]. This flexibility enables the integration of diverse sensing moieties for specific detection tasks. Furthermore, the morphology and composition of nanofibers: diameter, porosity, alignment, or core–shell configuration can be finely tuned by regulating electrospinning parameters including voltage, flow rate, and collector distance, as well as solution properties such as viscosity, conductivity, and surface tension [18]. Such control allows precise optimization of sensor performance for targeted applications. In addition, the mechanical flexibility of electrospun mats allows them to conform to curved or irregular surfaces, supporting their incorporation into flexible electronics, wearable systems, and food-packaging interfaces [19].

**Figure 1:**
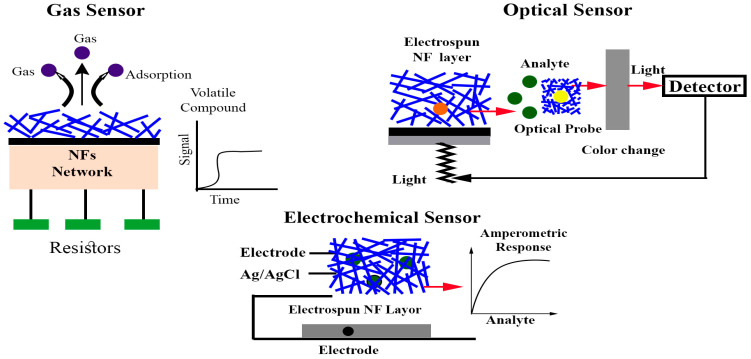
Schematic representation of major electrospun NF-based sensor type and their detection mechanisms.

Due to these unique advantages, electrospun nanofibers have emerged as a highly promising foundation for next-generation food sensors [18,19]. As a result, several recent reviews have examined their applications in food quality and safety monitoring [7,8,10,12,20]. However, despite extensive research on food sensing technologies, studies specifically focusing on electrospun nanofiber-based sensors for livestock-derived food quality assessment remain relatively scarce. The current review explores the materials, structural designs, and transduction mechanisms that reinforce nanofiber-based sensing platforms, emphasizing their application to real-time monitoring of livestock-derived food quality and safety. Particular attention is directed toward optical, electrochemical, and resistive mechanisms that translate physicochemical interactions at the nanoscale into measurable analytical signals (Figure 1). Studies from the food sector, especially meat quality and safety monitoring, are discussed to show practical performance metrics and industrial relevance. This review examines the role of electrospun nanofibers as active interfaces that enhance optical, electrochemical, and resistive sensing performance. The review further assesses current challenges and outlines future research directions aimed at advancing nanofiber-based sensor technologies toward scalable, sustainable, and intelligent food-monitoring systems.

## 2. Meat Quality Deterioration & Markers

Meat quality deterioration is primarily caused by microbial growth, enzymatic activity, and chemical reactions such as oxidation and browning. Microorganisms, including *Pseudomonas*, *Enterococcus*, *Clostridium*, *Salmonella*, *Lactobacillus*, and molds like *Penicillium* and *Cladosporium* contribute significantly to spoilage, while lipid oxidation generates hydroperoxides and secondary aldehydes (e.g., malondialdehyde, hexanal) that degrade flavor and texture. Enzymes such as lipases and proteases (calpains, cathepsins) further accelerate fat and protein breakdown [21]. Intrinsic and extrinsic factors such as animal type, age, pre- and post-slaughter handling, chilling, additives, and storage conditions also influence spoilage progression and shelf life [21,22]. Several physicochemical markers are used to assess meat quality, including temperature, humidity, gas composition (O_2_, CO_2_), and pH changes. Volatile nitrogen compounds (e.g., ammonia, dimethylamine, trimethylamine), biogenic amines (putrescine, cadaverine, histamine), and organic acids serve as biochemical indicators of microbial activity [23]. Analytical and sensing methods such as FTIR, LC–MS/MS, GC–MS, and time-temperature or humidity sensors integrated with RFID systems enable real-time monitoring of these markers, providing valuable insights into meat freshness, spoilage kinetics, and safety [24].

## 3. Electrospinning Process Overview

Electrospinning is a widely used technique that applies an electric field to a polymeric solution or melts to generate ultrafine fibers with nanoscale dimensions [25]. As the voltage increases, electrostatic forces gradually overcome the surface tension of the polymer droplet at the needle tip, causing it to deform into a Taylor cone, a characteristic conical shape that marks the onset of fiber formation [11,25]. Once the threshold voltage is reached, a charged jet is ejected from the cone and elongated in the electric field while the solvent evaporates, ultimately forming solid nanofibers collected as a non-woven web. Different electrospinning configurations, such as single-needle, multi-needle, needleless, coaxial, and multi-jet systems, have been developed to enhance throughput and tailor fiber structure [11]. In addition, other fabrication routes, including self-assembly, interfacial polymerization, and spinneret-based tunable engineered parameters (STEPs), have also been explored to produce nanofibers without high voltage [11]. Electrospinning performance is influenced by operating parameters such as applied voltage, flow rate, and needle/collector distance, which must be adjusted according to polymer properties and environmental conditions. Previous studies have reported optimization strategies for achieving uniform nanofibers [10,26].

## 4. Materials for Electrospun Nanofiber Sensors

The selection of a suitable polymer is crucial in the fabrication of electrospun nanofiber (NF) sensors, as it governs the resulting material’s biocompatibility, stability, processability, and capacity for chemical modification. Polymers used in electrospinning are broadly divided into synthetic and natural types, although polymer blends are increasingly employed to combine complementary attributes such as mechanical strength, hydrophilicity, and functional group availability [10,26].

### 4.1. Synthetic Polymers

Synthetic polymers are favored for their excellent mechanical strength, consistent batch quality, and ease of electrospinning, providing robust frameworks for diverse sensing applications. This ease of electrospinning arises from the favorable rheological and physicochemical properties of certain polymers, such as optimal viscosity, sufficient molecular chain entanglement, and good solubility, which together enable stable jet formation and uniform fiber deposition without bead defects. Moreover, their dielectric constant, charge mobility, and molecular polarity play critical roles in determining signal transduction efficiency, electron transfer behavior, and the overall stability of nanofiber-based sensors [27].

Poly(vinyl alcohol) (PVA) is one of the most widely used electrospinning polymers due to its water solubility, biodegradability, and hydroxyl-rich structure that allows easy cross-linking and surface modification. PVA nanofibers serve as effective hosts for dye encapsulation or metal nanoparticle decoration, improving optical and catalytic properties [27,28]. In optical sensing, PVA’s hydrophilicity enhances analyte adsorption and stabilizes chromophores for better signal reproducibility. Biodegradable aliphatic polyesters such as poly(l-lactic acid) (PLLA) and polycaprolactone (PCL) provide mechanical strength and hydrophobicity suitable for controlled analyte release and stable operation in humid environments, making them ideal for packaging-integrated sensors [29,30]. Polyacrylonitrile (PAN), a precursor for conductive carbon nanofibers, forms graphitic domains upon carbonization, promoting charge transfer and low impedance for amperometric and voltammetric sensing [31]. Poly(vinylpyrrolidone) (PVP) and poly(ethylene oxide) (PEO) are also used as matrices or co-spinning agents to adjust solution viscosity and ensure homogeneous nanoparticle dispersion during electrospinning and calcination, thereby enhancing catalytic activity and sensor response [32]. Other synthetic polymers that have been used for electrospinning include polystyrene (PS), polyaniline (PANI), Poly(vinylidene fluoride) PVDF, and polyurethane (PU) [10,26,27].

### 4.2. Natural Polymers

Natural polymers have attracted growing interest due to their biocompatibility, biodegradability, and environmental sustainability, making them ideal for food-contact sensing applications. Beyond their eco-friendly nature, their functional groups and physicochemical properties enhance sensor performance by promoting analyte adsorption, charge transfer, and molecular recognition. Chitosan, a deacetylated form of chitin, offers intrinsic antimicrobial activity and abundant amino and hydroxyl groups for enzyme or antibody immobilization [33]. Its protonable amino groups enable efficient electrostatic interactions and proton conduction, improving the sensitivity of electrochemical and impedance-based biosensors, while its cationic nature ensures strong adhesion and stable, uniform film formation on negatively charged substrates.

Cellulose and its derivatives, such as bacterial cellulose and cellulose acetate, exhibit excellent mechanical strength, hydrophilicity, and abundant hydroxyl groups that promote hydrogen bonding with sensing agents or cross-linkers [34,35]. Their semicrystalline structure ensures robustness, while polar hydroxyls facilitate analyte diffusion and uniform dye distribution in optical sensors, enhancing stability and colorimetric reversibility. Protein-based polymers like zein, gelatin, and silk fibroin offer sustainable, biocompatible nanofiber matrices. Gelatin contains natural cell-binding sites that support biomolecule attachment without denaturation, zein’s hydrophobic domains help encapsulate volatile compounds, and silk fibroin’s tensile strength and β-sheet crystallinity provide transparency, humidity resistance, and durability [36,37]. Additional natural polymers such as pullulan, chitin, alginate, and nitrocellulose have also been electrospun or used as nanofibrous substrates for food-contact sensors due to their hydrophilicity and functional group availability, supporting strong bioreceptor attachment and gas permeability [8,10,11].

### 4.3. Composite and Functionalized Fibers

The parameters of the electrospinning process can be adjusted to produce nanofibers with desirable configurations and performance characteristics. However, further enhancements are often necessary to achieve high sensitivity, selectivity, and stability in sensing applications. To accomplish this, electrospun nanofibers can be functionalized using various modification approaches. Chemical functionalization methods such as grafting, chelation, and crosslinking introduce reactive groups or recognition elements that improve analyte interaction. In contrast, physical functionalization approaches, including plasma treatment, ion beam implantation, and vapor deposition, modify the fiber surface or deposit active sensing materials without altering the underlying fiber structure [38,39]. Functionalization of electrospun nanofibers with metallic nanoparticles (e.g., Au, Ag) or metal oxides (e.g., TiO_2_, ZnO) can greatly enhance sensor performance by improving electrical conductivity, catalytic reaction sites, and plasmonic activity. These enhancements enable stronger signal generation and broaden the range of detection mechanisms [40,41].

Functionalization can be achieved via pre-electrospinning, co-electrospinning, and post-electrospinning. Nanofiber functionalization can occur at different stages of fabrication. Pre-functionalization modifies the polymer before spinning, while blend/co-electrospinning incorporates functional additives directly into the spinning solution. Post-functionalization, the most common approach, introduces sensing elements onto the fiber surface after fabrication, offering greater flexibility in material selection [42]. Core–shell nanofibers, fabricated via coaxial electrospinning, protect embedded dyes or biomolecules from leaching and photodegradation while allowing controlled analyte diffusion and sustained signal stability [43].

### 4.4. Mechanical and Structural Stability

The mechanical properties of electrospun nanofibers are largely governed by processing parameters and material characteristics. According to Al-Abduljabbar and Farooq (2023) [44], solution viscosity, molecular weight, and electrical conductivity determine whether the polymer jet forms continuous, bead-free fibers or disintegrates into droplets. Adequate chain entanglement and surface-tension control ensure structural continuity, while molecular alignment during electrospinning draw ratios up to 10^4^ and strain rates of 10^5^ s^−1^ enhance tensile strength compared with bulk films [44]. Conversely, low molecular weight or excessive voltage promotes bead formation and weak points. Friable or brittle fibers are particularly concerning for food-contact sensors, as fragmentation can generate microplastic debris, compromising both product safety and environmental integrity. While studies have shown that synthetic fibers may fragment and generate microplastics [45], the consequences of such particle release from electrospun sensors in food-contact applications remain largely unexamined. However, post-treatments such as thermal annealing, polymer blending, or PAN carbonization improve robustness and reduce friability, whereas careful selection of polymer composition (synthetic or natural polymers) directly affects flexibility, stability, and overall sensor reliability [44].

## 5. Optical Sensors for Visual and Spectral Analysis

Among the various sensing systems under development, optical sensing (Table 1) is regarded as one of the most promising approaches due to its simplicity, high specificity, and rapid response [46,47]. The major types of optical sensors are based on colorimetry, fluorescence, surface plasmon resonance (SPR), surface-enhanced Raman scattering (SERS), and combinations thereof. While each of these approaches offers distinct advantages, SERS is primarily suited for molecular fingerprinting, colorimetric sensors for on-site screening, fluorescent sensors for trace-level analysis, and SPR for real-time monitoring [46,47].

### 5.1. Colorimetric Freshness Indicators

Colorimetric sensors have attracted considerable attention because of their ability to provide rapid detection, simple operation, and easily observable responses that can be interpreted with the naked eye [46]. Colorimetric NF sensors provide direct visual indicators of food freshness through pH-dependent color transitions of natural dyes such as anthocyanins, curcumin, and shikonin, which respond to volatile amines produced during spoilage. These sensors operate by reacting with metabolites or target molecules (e.g., NH_4_, CO_2_, H_2_S, organic and biogenic amines), producing distinct color variations [8,48]. The electrospun architecture enhances dye stability and responsiveness by preventing aggregation and promoting uniform analyte exposure. The porous fiber network accelerates volatile transport and facilitates local proton exchange, enabling more rapid and reversible chromatic shifts [49,50]. In a study, Sun et al. developed a poly(l-lactic acid) (PLLA)/anthocyanin NF label that exhibited distinct color changes from pink to colorless as mutton spoiled, demonstrating faster response kinetics than dense films due to improved mass transfer (Table 2) [29].

In another study, Duan et al. reported dual-dye pullulan/chitin NFs containing anthocyanin and curcumin, extending the dynamic detection range and allowing discrimination of multiple freshness stages in fish [51]. These types of nanofiber-based colorimetric platforms combine environmental sustainability, visual clarity, and rapid responsiveness, making them ideal candidates for intelligent packaging and real-time freshness monitoring. Recently, a polyacrylonitrile nanofiber mat (PAN NFM)-based colorimetric sensor array (CSA) embedded with multiple pH-responsive dyes was developed for rapid and intelligent monitoring of fish freshness. The highly porous PAN structure facilitated efficient gas diffusion and achieved visible color transitions within 30 s upon exposure to volatile amines. By integrating machine learning and deep learning models, the CSA enabled non-destructive prediction of total volatile base nitrogen (TVB-N) and achieved 100% accuracy in classifying freshness stages, demonstrating its potential as a smart platform for real-time food quality assessment [52].

Several studies have also utilized NFs in colorimetric sensors for the detection of pathogens [46]. In a study by Zhang et al. (2023), a nanofibrous membrane (NFM) platform was developed for the detection of bacteria [58]. NFs were selected due to their porous nature, which is lacking in materials such as polymer membranes, filter paper, hydrogel or glass. Functional nanofibrous membranes were utilized as a fabric-based colorimetric sensor for the detection of *Escherichia coli.* During metabolism, β-glucuronidase was produced by *E. coli*, which caused the functionalized NFM to generate biological signals and color alterations. The images were analyzed using a smartphone app. The nanofiber-based platforms had a limit of detection (LOD) for *E. coli* at 26 CFU mL^−1^ in 15 min. Furthermore, these rapid analyses could be integrated into a smartphone app [58]. Nonetheless, electrospun NF-based colorimetric sensors provide unique merits that include low cost, rapid visual response, and compatibility with intelligent packaging systems. Their porous architecture enhances dye stability and gas–fiber interactions, enabling faster and more sensitive spoilage detection than bulk films. However, most reported platforms are still qualitative or semi-quantitative, and their performance may be affected by lighting conditions, humidity, and dye leaching.

### 5.2. Fluorescent Sensors

**Figure 2:**
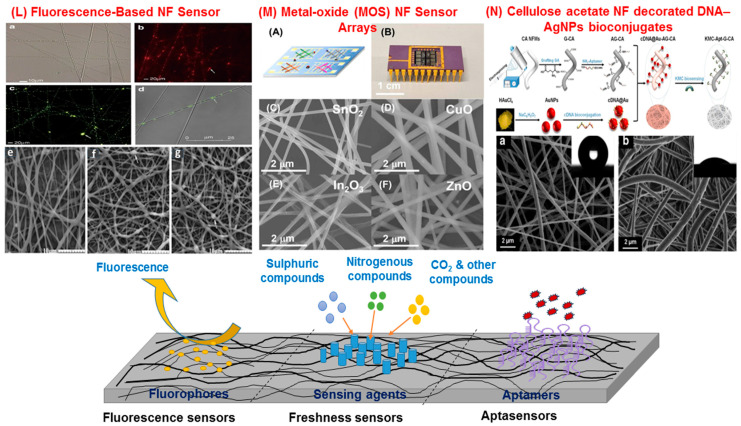
Multifunctional nanofiber (NF)-derived materials. (*L*) Fluorescence-based NF sensor: electrospun composite NFs containing chitosan (CS) nanoparticles (NPs); (**a**) optical image; (**b**) fluorescence image of rhodamine B-loaded fibers; (**c**) fluorescence image of FITC-labeled NPs within fibers; (**d**) laser scanning confocal microscopy image; SEM images of (**e**) NFs without NPs, (**f**) composite NFs with rhodamine B-loaded CS NPs, and (**g**) composite NFs with naproxen-loaded CS NPs. (*M*) Metal-oxide (MOS) NF gas sensor arrays: (**A**) conceptual illustration of the sensor array exposed to analytes; (**B**) photograph of the array mounted on a DIP chip carrier via wire bonding; (**C**–**F**) SEM images of electrospun NFs composed of (**C**) SnO_2_, (**D**) CuO, (**E**) In_2_O_3_, and (**F**) ZnO. (*N*) Cellulose acetate (CA)-based biosensor: schematic illustration of CA, G-CA, and AG-CA NFM fabrication, AuNP–cDNA bioconjugation, cDNA@Au assembly, and KMC biosensing process; representative FE-SEM images of (**a**) CA NFM and (**b**) G-CA NFM. (Adapted from Wang et al. 2011 [60]; Abedalwafa et al., 2020 [62]; Zang et al., 2023 [63]).

Fluorescent sensors are based on the utilization of fluorophores to absorb light at certain wavelengths and emit light at longer wavelengths (Figure 2). This is mainly due to the excited state of the fluorophore and its interaction with its surroundings. While these sensors use diverse materials, commonly used fluorophores (organic) include rhodomine, fluorescein and cyanines. Other fluorophores investigated include Inorganic phosphors, Green Fluorescent Protein (GFP), and nanomaterials (quantum dots, carbon dots, silica nanoparticles). While GFP is a naturally derived protein that can provide real-time monitoring, quantum dots offer size-tunable fluorescence as well as superior brightness. NMs, such as carbon dots and silica nanoparticles, have superior photostability. Fluorophores can also be integrated with metallic nanoparticles to enhance brightness and sensitivity. Fluorescent sensing mechanisms include fluorescence quenching, where analyte interaction reduces emission intensity. Molecular recognition, where probe–analyte binding alters fluorescence for selective detection and Fluorescence Resonance Energy Transfer (FRET), involving energy transfer between donor–acceptor fluorophores for highly sensitive analysis. Additionally, pH-sensitive fluorophores enable monitoring of pH-dependent biochemical changes in complex samples [59]. However, NFs have been used to improve the performance of fluorescent sensors. NFs prevent aggregation-induced quenching and improve photostability by isolating fluorophores within a porous and optically active network. This enhances local excitation efficiency and enables dynamic ratiometric detection through spatially resolved emission. In a study, Quan et al. developed cellulose nanofiber-based ratiometric fluorescent sensors by grafting fluorescein isothiocyanate (FITC) and incorporating protoporphyrin IX as an internal reference for biogenic amine detection (LOD = 1 ppm). Although the fibers were prepared via TEMPO oxidation rather than electrospinning, the study demonstrates that fibrous polymer networks can enhance light transmission, gas diffusion, and fluorescence stability [53]. However, in an earlier study by Wang et al. [60], Fluorescein isothiocyanate (FITC) was incorporated within the chitosan–tripolyphosphate nanoparticles forming the fiber core, whereas Rhodamine B was localized in the polycaprolactone shell. The resulting core–shell nanofibers exhibited distinct dual fluorescence, confirming spatial separation of the dyes. This study also showed that the core environment preserved fluorescence stability while the shell region provided stronger initial emission intensity [60]. In a study by Li et al. (2019), polystyrene (PS) based electrospun NFs integrated with NaYF_4_:Yb^3+^ and Er^3+^ upconversion nanorods (serving as fluorophores) and Au@SiO_2_ plasmonic nanostructures were developed to achieve enhanced fluorescence emission [54]. The fibrous matrix provided a flexible, high-surface-area scaffold for uniform dispersion of the luminescent nanorods, while the plasmonic components amplified the upconversion signal. These upconversion–plasmonic nanofiber composites demonstrate exceptional sensitivity for detecting trace contaminants (Rhodamine B) and hold strong potential for optical sensing applications in food safety monitoring [54].

In addition to high surface area, the mechanical flexibility of polymers improves contact with substrates and enhances the durability of sensing films [60]. The interconnected porous architecture of electrospun fibers also regulates analyte permeability and diffusion, ensuring rapid mass transfer and efficient interaction with embedded functional materials [52,53]. Moreover, polymers with tailored dielectric properties and ionic conductivity can facilitate electron or ion transport, stabilize charge-transfer interfaces, and modulate fluorescence responses in nanocomposites [54]. Although these nanocomposites have not yet been evaluated as nanosensors for meat quality assessment, they hold significant potential for such applications. Nevertheless, Nanofiber-based fluorescent sensors provide high sensitivity and fast responses due to efficient light interaction and mass transfer within the porous fiber structure, making them promising for real-time freshness monitoring. However, selectivity can be limited by overlapping emission profiles, and fluorescence signals are often influenced by humidity, temperature, and pH. Ensuring fluorophore stability and preventing leaching also remain challenges [61]. Therefore, further improvements in robustness and quantitative performance are needed before widespread application in meat quality assessment.

### 5.3. SERS Substrates for Trace Contaminant Detection

**Figure 3:**
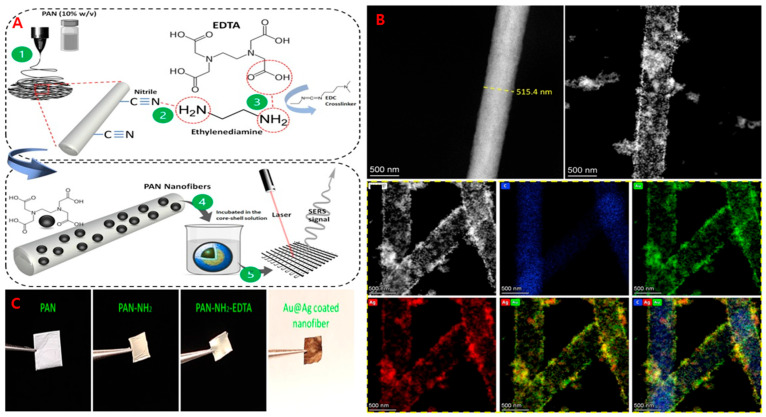
(**A**) Schematic diagram of surface functionalization of polyacrylonitrile electrospun nanofibers: (1) electrospinning of PAN nanofibers; (2) amine functionalization; (3) EDTA grafting via EDC crosslinking; (4) synthesis of Au–Ag core–shell nanoparticles; and (5) deposition of Au@Ag NPs onto EDTA-modified PAN NFs. (**B**) TEM image showing NF before and after surface modification. (**C**) Various phases of functionalization, immobilization, and coating of the substrate. Adapted from (Hajikhani et al., 2024 [55]).

Surface-enhanced Raman scattering (SERS) is an advanced form of Raman spectroscopy that greatly increases detection sensitivity, allowing analyte identification at parts-per-billion levels [64]. Porous, metal-functionalized substrates with high surface area have been reported to be critical for amplifying Raman signals. However, electrospun nanofiber scaffolds are specifically suitable for this purpose owing to their large surface-to-volume ratio and tunable morphology. Electrospun NFs serve as SERS substrates, providing a supporting framework on which metallic nanoparticles (Ag, Au, or Cu) are deposited [55,65]. Recent studies show that integrating electrospinning with SERS enhances analytical performance, enabling precise detection of microorganisms such as *E. coli* and *S. aureus*, as well as various organic molecules, pesticides, herbicides, and chemical residues [55]. In an investigation by Hajikhani et al. (2024), the substrate used was an electrospun polyacrylonitrile (PAN) nanofiber mat, sequentially amine- and EDTA-functionalized, and finally coated with gold–silver (Au@Ag) core–shell nanoparticles [55]. Figure 3 illustrates the surface functionalization of the PAN nanofiber mat. Electrospun NFs were used as a substrate to improve the efficacy of contaminant detection. This nanofiber–plasmonic hybrid substrate served as the SERS-active platform for detecting thiabendazole in soy milk with high sensitivity. The limits of quantification (LOQ) were found to be ~70 ppb [55]. Although a negligible amount of thiabendazole can be found in meat, sensitive SERS-based screening methods remain essential for routine surveillance.

Incorporating plasmonic nanostructures (Au, Ag) into electrospun NFs integrates the electromagnetic amplification of SERS with the large surface area and mechanical flexibility of the fibrous substrate. The nanoscale junctions between embedded metal particles generate dense electromagnetic hotspots, intensifying Raman scattering and enabling single-molecule sensitivity. Sarma et al. developed AuNP-decorated electrospun PVA fibers for detecting antibiotic residues such as doxycycline and enrofloxacin in chicken meat, achieving parts-per-billion (ppb) sensitivity validated by LC–MS [56]. Similar approaches have identified banned additives, including malachite green in fish and melamine in milk [66,67]. The incorporation of metallic nanomaterials onto nanofibers can also be achieved through an ex situ decoration approach. For instance, Chen et al. (2022) developed a SERS substrate by first electrospinning polyvinyl alcohol (PVA)–polyethyleneimine (PEI) nanofibers, followed by electrostatic adsorption of silver nanoparticles (AgNPs) onto their surface [28]. The resulting AgNP-decorated PVA–PEI nanofibers exhibited excellent SERS activity and were successfully applied for the detection of enrofloxacin in prawn samples. Enrofloxacin, a fluoroquinolone antibiotic used in aquaculture, along with furazolidone and malachite green, is toxic and promotes antimicrobial resistance. Hence, these drugs are banned in many countries but still used in some developing regions [28]. Zhang et al. demonstrated zein-based NFs coated with Ag nanoparticles via electrospraying for nitrite detection in cured meats, achieving reproducible enhancement and superior hotspot uniformity [57]. Nitrites, widely used in cured meats for their antimicrobial action against *Clostridium botulinum*, are monitored due to their potential to form carcinogenic nitrosamines linked to colorectal and gastric cancers [8]. These SERS-integrated NF substrates exemplify the way in which plasmonic coupling and interfacial stabilization within biopolymer matrices can yield flexible, portable, and ultra-sensitive optical sensors for real-world food diagnostics.

## 6. Electrochemical Sensors for Precise Quantification

**Figure 4:**
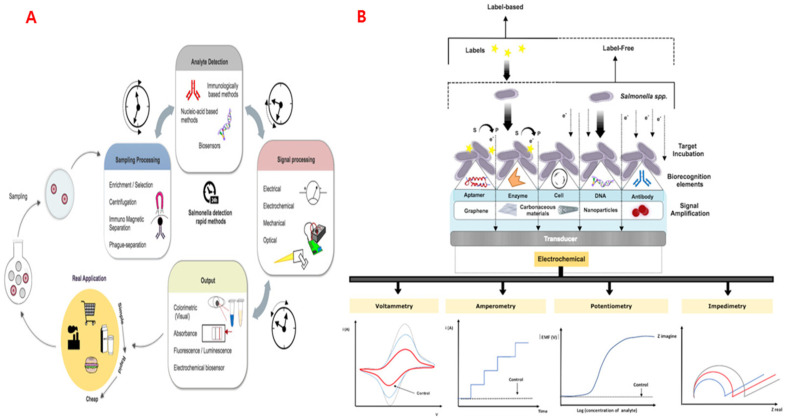
(**A**) Schematic diagram of current rapid methods for pathogen detection in food products. (**B**) Schematic representation of typical biosensor elements (transducer, amplification layers, bioreceptor), with different detection modes (label-based or label-free) and electrochemical transducing techniques (voltammetry, amperometry, potentiometry and impedimetry). Label-based biosensors employ signal tags (★) such as enzymes, redox mediators, or nanoparticles to amplify the electrochemical signal. The red curves in voltammetric and impedimetric plots represent the response after *Salmonella* binding, showing increased current or impedance relative to the control (black curve). Adapted from Silva et al., 2018 [70].

Electrochemical sensors, a subset of chemical sensors, utilize electrodes as transducers to monitor chemical variations within a system. Based on electrochemical principles, these sensors identify and quantify specific analytes by translating their interactions with an electrode surface into measurable electrical signals. However, it is important to distinguish such analyte-specific sensors from systems like the electronic tongue, which generate holistic electrochemical fingerprints of complex sample matrices rather than quantifying individual components [68]. Electrochemical biosensors are broadly categorized into amperometric/voltametric, potentiometric, and conductometric types, depending on the electrical parameter measured. In amperometric sensors, the signal arises from the current generated during oxidation–reduction reactions. Potentiometric sensors determine the voltage difference between a working and reference electrode, while conductometric sensors evaluate variations in a sample’s electrical resistance or conductivity [69]. To improve sensor performance, support materials have been integrated with nanoscale components, providing additional structural and functional benefits. Materials widely used as biorecognition elements include enzymes, aptamers, DNA, and antibodies (Figure 4). Nonetheless, various nanomaterials are utilized in electrosensors to not only improve receptor immobilization but also amplify signals [70].

### 6.1. Carbon Nanofiber (CNF) Electrodes

Among the most prominent electrospun architectures used in electrochemical sensors are carbon nanofibers (CNFs), which are typically produced by the pyrolysis of electrospun polyacrylonitrile (PAN) mats. The resulting CNFs are freestanding, mechanically robust, and electrically conductive, making them ideal scaffolds for electrode fabrication. Their one-dimensional morphology and interconnected networks provide continuous electron pathways and high surface roughness for improved catalyst loading and signal amplification. The high aspect ratio facilitates rapid charge transfer by shortening electron pathways between redox sites and electrodes [71].

In a study, Ahmad et al. (2022) developed amperometric sensors using cobalt(benzene-1,3,5-tricarboxylic acid) metal–organic framework [Co(TMA)MOF] deposited onto electrospun porous carbon nanofibers (NFs) via a solvothermal method [71]. The resulting sea-urchin-like Co(TMA)MOF–carbon NF electrodes effectively detected xanthine and uric acid in salmon fillet, with detection limits of 96.2 nM and 103.5 nM, respectively. In various seafood species, including fish and shellfish, the concentrations of adenosine triphosphate (ATP) and its degradation products, such as xanthine, hypoxanthine, and uric acid, are commonly used as biochemical markers of freshness (Table 3). However, in this study, the polyacrylonitrile (PAN)-derived carbon NFs (280–300 nm diameter) provided a 3D porous architecture that enhanced analyte adsorption and electron/ion transfer, improving electrocatalytic activity. This study showed the additional advantages of electrospun carbon NFs, including flexibility, recyclability, and high porosity, which make them ideal platforms for enzyme immobilization and electrochemical sensing [71]. The synergistic interaction between the conductive CNF substrate and the catalytically active MOF nanoparticles enabled ultralow detection limits in the nanomolar range and stable performance across multiple cycles. Similarly, Lim and Ahmed utilized electrospun CNFs as a platform for antibody immobilization, creating an immunosensor capable of detecting porcine serum albumin as a biomarker of pork adulteration in meat products. Due to the higher cost of meats such as beef and mutton, cheaper meats like pork are sometimes used for adulteration. To identify these adulterants, sensitive and cost-effective sensors are being studied [72]. Nonetheless, the resulting sensors have exhibited high specificity, repeatability, and fast response, illustrating the potential of CNF-based electrodes for ensuring food authenticity and traceability. CNF-based electrodes offer high conductivity, large surface area, and excellent electrocatalytic performance, enabling sensitive detection of meat freshness markers and adulteration indicators [71]. However, high-temperature processing and interference from complex food matrices remain challenges. Further optimization is needed to improve long-term stability and support broader practical deployment.

### 6.2. Enzyme-Based Sensors

Enzyme-based biosensors rely on the high substrate specificity and catalytic efficiency of enzymes to detect certain metabolites or contaminants through measurable electrochemical responses [64,70]. NF morphology enhances enzyme–substrate contact area and improves electron mediation between enzyme cofactors and electrodes. Furthermore, electrospun NF mats provide a favorable environment for enzyme immobilization due to their large surface area, mechanical stability, and ability to preserve enzyme activity through mild attachment chemistries. In a study to evaluate the freshness of chilled seafood such as squid and large yellow croaker, Wang et al. [64] developed an amperometric electrochemical sensor incorporating xanthine oxidase (XOD) immobilized on copper-based metal–organic framework nanofibers (CuMOF NFs). The MOF nanofibers, formed by coordinating organic ligands with copper salts, were combined with sodium alginate to produce stable films enabling efficient enzyme entrapment and electron transfer. The resulting XOD–CuMOF sensor exhibited excellent sensitivity for hypoxanthine and xanthine detection, with detection limits of 0.0023 μM and 0.0064 μM, respectively, demonstrating strong potential for rapid seafood freshness monitoring [64].

Beyond freshness monitoring, enzyme-functionalized NFs have also been applied for detecting harmful residues. Acetylcholinesterase (AChE), for instance, can be immobilized on electrospun polymer nanofibers to detect organophosphate and carbamate pesticides through enzyme inhibition mechanisms [70]. The decrease in enzymatic activity correlates with pesticide concentration, providing a simple and sensitive approach for assessing food contamination. These studies indicate the adaptability of electrospun nanofiber sensors for multiple enzymatic detection modes. Enzyme-based nanofiber sensors offer excellent selectivity and very low detection limits for freshness markers and pesticide residues. Yet enzyme activity can be influenced by environmental conditions and food matrix interference, which may limit long-term stability and practical application.

### 6.3. Aptamer-Based Sensors

Label-free electrochemical sensors, such as impedimetric and aptamer-based systems, have gained considerable attention for their exceptional sensitivity and ability to detect binding events directly on the electrode surface without requiring secondary labels or enzymes. Impedimetric sensors detect variations in electrical impedance that occur as a result of biochemical interactions, such as the attachment of target biomolecules to specific bioreceptors immobilized on the sensor surface [75]. Among the various bioreceptors, aptamers have emerged as superior substitutes for the traditional utilization of antibodies as biorecognition material. Antibodies have been used as biorecognition elements in biosensors because of their strong affinity and specificity toward various analytes. However, their use is limited by factors such as sensitivity to chemical modification, thermal instability, and the high cost and complexity of production. In contrast, aptamers, which are single-stranded DNA or RNA molecules, offer a simpler, in vitro selection process and exhibit high affinity, selectivity, and sensitivity toward target analytes [76].

Electrospun nanofibers offer an advantageous interface for these sensors by providing increased electrode roughness and a high density of reactive sites for probe immobilization, resulting in amplified impedance or resistance shifts upon analyte binding. In an earlier study, Thiha et al. constructed a sensor using electrospun carbon nanowires functionalized with a *Salmonella*-specific aptamer [73]. The flexible nanowire network acted as a transducing element that translated aptamer–pathogen interactions into measurable electrical resistance changes. The sensor achieved an impressive detection limit of 10 CFU/mL within just 5 min, indicating its potential for rapid on-site pathogen detection. This principle can readily be extended to other foodborne bacteria, including *Listeria monocytogenes* and *Escherichia coli* O157:H7, enabling broad-spectrum microbial surveillance in complex food matrices [73]. In an earlier study, a conductometric sensor employing antibodies was developed to detect *E. coli* O157:H7 and bovine viral diarrhea virus (BVDV). The electrospun nitrocellulose nanofiber capture pad provided a large surface area for antibody immobilization, significantly improving pathogen capture and detection [6]. However, in another study, *Salmonella* was detected in milk using an aptasensor with a chitosan–carbon nanofiber and gold nanoparticle-modified graphite electrode. The CNF sensor exhibited enhanced sensitivity, achieving a detection limit of 1.223 CFU/mL, far lower than PCR’s 10^2^ CFU/mL [74]. *Salmonella*, a rod-shaped Gram-negative bacterium from the *Enterobacteriaceae* family, causes an estimated 1.4 million foodborne infections annually in the United States [73]. In another study by Yao et al. (2020), a novel nanocomposite was fabricated that consisted of mine-functionalized metal–organic framework (UiO-66-NH_2_), and a multiwalled carbon nanotube@reduced graphene oxide nanoribbon (MWCNT@rGONR) [77]. The UiO-66-NH_2_/MCA/MWCNT@rGONR nanocomposite was developed as an electroactive platform for aptamer-based detection of kanamycin, leveraging its high surface area, rich amino functionality, and superior conductivity. The aptasensor showed excellent accuracy and reliability in detecting kanamycin in animal-derived foods, achieving recoveries of 98.3–107.7% in fish and 97.8–103.7% in milk with low RSD values (2.01–4.86%). Although the aforementioned studies have utilized carbon nanowires and nanotubes, similar carbon NFs based nanocomposites still need to be investigated.

Recent studies have shown that electrospun nanofibrous membranes (NFMs) enhance sensor sensitivity and portability due to their high surface area, porosity, and ease of functionalization. Their interconnected porous structure promotes efficient analyte diffusion and binding. However, while NFMs have been explored separately with AuNPs for photocatalysis and aptamers for electrochemical sensing, the integration of aptamer–AuNP bioconjugates with NFMs for colorimetric antibiotic detection remains largely unexplored. Therefore, Abedalwafa et al. (2020) fabricated a portable sensor strip based on aptamer-functionalized electrospun nanofiber membranes and DNA–AuNP probes for kanamycin detection [62]. The sensor exhibited a distinct color change from pink to white upon target binding, with a detection limit of 2.5 nM and excellent recoveries (98.9–102.2%) in drinking water and milk, demonstrating strong potential for rapid food safety monitoring. In another study, a highly sensitive electrochemical aptasensor was developed for ochratoxin A (OTA) detection in cold brew coffee using silanized cellulose nanofibrous membranes functionalized with methylene blue-labeled aptamers [78]. In some countries, OTA, a mycotoxin, has been detected in meat and meat products. OTA is reported to show carcinogenic and immunotoxic effects in both animals and humans and is categorized as possibly carcinogenic to humans [79]. However, the nanofiber-modified electrode doubled the active surface area, achieving an ultralow detection limit of 0.81 pg mL^−1^ and enabling direct, stable, and reusable OTA detection without sample pretreatment [78]. While the EU has not established maximum OTA levels in meat and meat products, some EU nations (Denmark, Estonia, Romania, Slovakia and Italy) have included regulations or guidelines on OTA concentrations in meat and meat products [79]. Nonetheless, aptamer-based nanofiber-derived sensors show strong potential for meat quality monitoring due to their high sensitivity, rapid response, and compatibility with portable formats. These sensors support reliable detection of pathogens and contaminants directly in meat samples. Continued improvements in stability will help advance these platforms toward practical use in real-world meat safety testing.

## 7. Resistive Gas Sensors (Electronic Noses)

Resistive gas sensors, commonly integrated into electronic nose (E-nose) systems, function by measuring changes in electrical resistance upon exposure to volatile compounds. These sensors are designed to mimic the olfactory response of biological systems, generating a distinctive electrical fingerprint corresponding to complex odor mixtures. These types of devices are mainly useful for comprehensive quality assessment and freshness grading in food products, as they provide holistic information rather than targeting a single analyte. Thus, employing an electronic nose (e-nose) for meat quality monitoring offers an efficient approach to detect spoilage and prevent the consumption of degraded meat, while also addressing issues related to unpleasant odor emissions [80].

Electrospinning enables the fabrication of metal oxide semiconductor (MOS) nanofibers such as SnO_2_, ZnO, and TiO_2_ with high surface area and abundant active sites for gas adsorption (Table 4). In a recent study, Ag- and Pt-decorated SnO_2_ nanowire arrays demonstrated enhanced catalytic activity and selectivity for H_2_ and NH_3_, achieving detection limits of 0.2–1.2 ppm with rapid response and recovery times. The detection mechanism is based on changes in the electrical resistance of SnO_2_ when gas molecules interact with its surface. The signal enhancement was due to the synergistic effect of the SnO_2_ nanowire structure and Ag/Pt nanoparticle decoration. The nanowires ensured efficient charge transport, while the metals introduced catalytic and electronic sensitization. This combined effect amplifies the gas response, thereby improving sensitivity and selectivity [81].

Andre et al. developed hybrid free-standing SiO_2_:In_2_O_3_, SiO_2_:ZnO, and SiO_2_ nanofiber mats modified with polyaniline and poly (styrene sulfonate) as sensing platforms for detecting volatile amines (ammonia, methylamine, trimethylamine) released during meat and fish spoilage. The detection mechanism relies on impedance changes caused by the interaction of volatile amines with the conductive polymer layer. The SiO_2_–metal oxide nanofibers facilitate efficient gas adsorption and charge transfer, while the polyaniline coating enhances conductivity and sensitivity, enabling selective and stable detection of spoilage-related amines in fish samples [82]. Nonetheless, the NF-based systems are known to successfully discriminate between varying stages of freshness by analyzing the complex volatile signature emitted during storage. These types of electrospun MOS-based E-noses can be calibrated to recognize characteristic odor profiles across a wide spectrum of foods, including dairy, grains, meats, and beverages. By correlating the sensor array’s resistance pattern with trained data models, these systems can deliver real-time, non-destructive freshness grading and shelf-life estimation. Similarly, Zang et al. [63] developed a multicomponent E-nose composed of electrospun SnO_2_, CuO, In_2_O_3_, and ZnO nanofiber sensors, each exhibiting uniform morphology with an average fiber diameter of 150 nm. The detection mechanism was based on resistance modulation in response to gas adsorption on the metal oxide surface. The E-nose array effectively discriminated five volatile organic compounds (VOCs), ammonia, ethanol, acetaldehyde, isoprene, and acetone, through principal component analysis (PCA) of sensor response patterns. This system demonstrated high sensitivity and selectivity, showing potential for real-time and non-invasive food quality monitoring [63].

## 8. Functional Roles and Comparative Performance of Electrospun NF-Based Sensors

Studies have shown that electrospun NFs can either form the active sensing matrix itself or serve as a high-surface-area scaffold that enhances immobilization, electron transfer, and optical signal amplification (Table 5) [64,71,73]. Depending on the incorporated materials such as metal–organic frameworks, plasmonic nanoparticles, or biorecognition elements, these nanofibers exhibit outstanding sensitivity and selectivity across optical, electrochemical, and resistive sensing modalities [54,55,63,73,78,81,82].

Nanofiber-based sensing platforms demonstrate distinct advantages depending on application needs. Colorimetric NF sensors remain the most practical for innovative packaging due to their low cost, scalability, and visual readout, although their largely qualitative nature and susceptibility to humidity and temperature variations limit precision (Table 6). Studies have shown that anthocyanin- or curcumin-based colorimetric nanofibers fabricated from PLLA [48], pullulan/chitin [51], and cellulose [35] provide rapid and reversible color responses toward spoilage-related volatile amines. Fluorescent NF sensors, such as those using FITC/protoporphyrin IX cellulose nanofibers [53], dual-fluorophore chitosan nanofibers [60], or upconversion–plasmonic PS composites [54], achieve high sensitivity and enable quantitative readouts. Electrochemical NF sensors, particularly those employing carbon nanofibers or aptamer functionalization, achieve ultralow detection limits and strong selectivity suitable for regulatory food safety testing. However, fabrication complexity and bioreceptor instability can increase cost. Some examples of NF-based electrochemical sensors include enzyme-based CuMOF [64] and CoMOF/carbon nanofiber [71] sensors for xanthine and hypoxanthine detection. Aptamer-modified nanofibers have resulted in the selective recognition of pathogens and toxins [73,78]. Resistive gas-sensing NF systems offer real-time, IoT-compatible spoilage detection but face drift and selectivity challenges in humid meat environments, partly due to reliance on metal-oxide semiconductors and noble-metal catalysts. Emerging research into biodegradable polymers, solvent-free electrospinning, and recyclable device formats is expected to enhance sustainability and industrial adoption across all sensor types.

## 9. Challenges and Future Perspectives

The development of on-site and on-package sensors that provide rapid, sensitive, and selective detection with minimal sample preparation and low cost remains a major challenge [83]. Multiplexed sensing platforms capable of identifying several spoilage or contamination markers are preferred to single-analyte systems, as not all pathogens or metabolites are equally hazardous [84]. Despite significant laboratory progress, industrial translation is still limited by production cost, reproducibility, and long-term stability [85]. Conventional nanomaterials such as carbon nanotubes and noble metals provide excellent sensitivity but raise concerns regarding sustainability and scalability [86]. Future efforts should therefore emphasize the development of affordable, durable, and environmentally benign materials that maintain stability under fluctuating humidity and temperature conditions [87]. Another critical issue is chemical migration from electrospun nanofibers used in food-contact applications. Although many electrospun biopolymers originate from natural sources and exhibit good biocompatibility, residual solvents, surfactants, and crosslinkers may remain in the fibers and migrate into food [88,89]. Such migration could pose health risks if not properly controlled. Therefore, migration and cytotoxicity assessments are required to ensure compliance with EU and FDA food-contact regulations [90,91]. The use of green solvents, low-toxicity additives, and solvent-free electrospinning approaches is strongly recommended to reduce potential contamination while maintaining processability and safety [91,92].

Conventional fabrication techniques such as chemical vapor deposition and lithography remain unsuitable for large-scale production of flexible sensors [26]. In contrast, needleless, coaxial, and triaxial electrospinning strategies have emerged as promising alternatives for scalable, uniform, and high-throughput nanofiber fabrication [93]. Future research should also focus on the development of biodegradable and intelligent electrospun sensors that can integrate seamlessly with IoT platforms, wireless readers, and AI-based data analytics [94,95]. The convergence of material innovation and digital technology will enable real-time, cloud-connected food-quality monitoring, facilitating the transition from laboratory-scale demonstrations to industrial applications.

## 10. Conclusions

Electrospun nanofibers have emerged as a transformative material platform for sensing technologies due to their exceptionally high surface area, tunable porosity, and versatile surface chemistry. These features address key limitations of conventional sensors and have enabled the development of optical, electrochemical, and resistive systems with enhanced sensitivity, faster signal transduction, and improved operational stability. In meat and seafood monitoring, nanofiber-based sensors have shown high potential for detecting spoilage markers, pathogenic bacteria, harmful residues, and adulterants with strong analytical performance.

From a practical standpoint, different nanofiber-based sensing platforms offer unique advantages and constraints. Colorimetric sensors provide low-cost visual readouts suitable for smart packaging but are mostly qualitative and affected by environmental variation. Fluorescent sensors deliver greater sensitivity and quantification but require optical instrumentation and controlled testing conditions. Electrochemical sensors, particularly CNF- and aptamer-functionalized systems, achieve ultralow detection limits and accurate quantification, although fabrication costs and bioreceptor stability remain concerns. Resistive gas-sensing nanofibers enable real-time monitoring and IoT integration but still face humidity-induced drift and selectivity challenges. Efforts toward biodegradable and solvent-free nanofiber systems are also advancing sustainability without compromising performance.

Despite their promise, large-scale manufacturing, long-term stability in complex food matrices, and regulatory approval remain important challenges. Ongoing progress in functional nanomaterials, scalable electrospinning systems, and miniaturized electronics is expected to accelerate translation from laboratory prototypes to commercial food safety solutions. Electrospun NF sensors hold significant potential to improve real-time meat quality assessment and contribute to broader fields such as environmental monitoring and clinical diagnostics.

## Figures and Tables

**Table 1:** Major types of NF-derived sensors (adapted from Akhavan-Mahdavi et al., 2024 [47]).

Sensing Mode	Mechanism/Feature
Optical	Detect variations in light intensity, wavelength, polarization, or decay characteristics caused by analyte interaction. Techniques such as fluorescence, phosphorescence, refraction, interference, and Raman scattering are commonly applied. Optical nanofiber sensors are valued for their high sensitivity, rapid response, and ability to enable real-time, non-destructive analysis.
Electrochemical	Operate by translating chemical or biochemical reactions into electrical signals (current, potential, or impedance). These platforms offer excellent sensitivity, low detection limits (often in the nanomolar to picomolar range), and cost-effective miniaturization. Widely applied in food safety and biomedical monitoring.
Fluorescent	Utilize fluorescent dyes, quantum dots, or fluorophore-tagged molecules that emit light upon excitation. Because of their high signal-to-noise ratio, such systems are ideal for detecting trace analytes or biomolecules even in complex media.
Colorimetric	Provide visible color transitions triggered by changes in pH, redox state, or volatile metabolite levels. The color shift serves as an immediate, on-site indicator of food freshness or environmental contamination without requiring external instrumentation.
Resistive	Measure alterations in electrical resistance or conductivity resulting from mechanical deformation, pressure, humidity, or gaseous exposure. These sensors are particularly suitable for strain, stress, and volatile organic compound (VOC) detection.
Photoelectric	Depend on the change in photo-induced current or voltage upon exposure to light or analytes that affect optical absorption. Commonly used for measuring material reflectance, transparency, or analyte-related optical variations.
Mass-Sensitive	Function through the adsorption of analytes on a piezoelectric or resonant surface, leading to measurable frequency or phase shifts. Quartz crystal microbalance (QCM) sensors are a typical example, allowing precise quantification of adsorbed mass.
Acoustic Wave	Rely on the modulation of surface or bulk acoustic waves when molecules interact with the sensor surface. The resulting frequency or phase shift correlates directly with analyte concentration, enabling sensitive gas or vapor detection.
Amperometric	Quantify the electric current generated during oxidation or reduction of electroactive species at the electrode interface. The measured current is proportional to analyte concentration, making this approach ideal for quantitative biosensing applications.

**Table 2:** Representative electrospun nanofiber–based optical sensors for meat quality and safety monitoring.

Nanofiber Composition	Sensor Type	Sensing Agent/Functional Element	Target Analyte or Freshness Marker	Application/Features	Detection Performance	Reference
PVA/PEI nanofibers + Ag NPs	SERS	AgNPs	Enrofloxacin (Prawn samples)	Sensitive antibiotic detection	LOD = ppb range	Chen et al., 2022 [28]
PLLA/Anthocyanin nanofibers	Colorimetric	Natural dye (anthocyanin)	Volatile amines in mutton	Color change pink → colorless; rapid response	Visual discrimination	Sun et al., 2021 [29]
Pullulan/Chitin nanofibers (anthocyanin + curcumin)	Colorimetric	Dual natural dyes	Volatile amines in fish	Differentiation of multiple spoilage stages	Visual discrimination	Duan et al., 2021 [51]
Polyacrylonitrile (PAN) nanofiber mat with multiple pH-responsive dyes	Colorimetric sensor array (CSA)	Diverse pH-sensitive dyes integrated within PAN nanofiber mat	Volatile amines and total volatile base nitrogen (TVB-N) in fish	Freshness prediction using ML/DL models; 100% accuracy in classifying freshness stages	LOD = 0.14 ppm Rapid color response within 30 s	Zhang et al., 2026 [52]
Cellulose nanofibers (FITC + protoporphyrin IX)	Fluorescent (ratiometric)	Fluorescein isothiocyanate (FITC) and protoporphyrin IX	Biogenic amines (seafood)	Ratiometric fluorescence sensor; visual color change correlated with spoilage	LOD = 1 ppm	Quan et al., 2021 [53]
Polystyrene (PS)/NaYF_4_:Yb^3+^,Er^3+^/Au@SiO_2_ nanofibers	Upconversion–plasmonic fluorescent	Upconversion nanorods (NaYF_4_:Yb^3+^,Er^3+^) and plasmonic Au@SiO_2_ nanoparticles	Rhodamine B (model contaminant)	Enhanced fluorescence due to plasmon–exciton coupling; high sensitivity for trace contaminant detection	LOD = 0.01 ppm. Small amount of sample required (≈10 µL)	Li et al., 2019 [54]
PAN nanofiber + Au@Ag nanoparticles	SERS	Au@Ag plasmonic NPs	Thiabendazole (soy milk)	High reproducibility and sensitivity	LOQ ≈ 70 ppb	Hajikhani et al., 2024 [55]
PVA Nanofibers	SERS	AuNPs	Detection of doxycycline, enrofloxacin (chicken samples	Sensitive antibiotic detection	LOD = ppb range	Sarma et al. (2023) [56]
Zein/Ag nanoparticle nanofibers	SERS	AgNPs	Nitrite in cured meat	Reproducible enhancement and hotspot uniformity	LOD = ppb range	Zhang et al., 2023 [57]

**Table 3:** Representative electrochemical nanofiber-based biosensors for meat and animal-derived products.

Nanofiber Composition	Sensor Type	Recognition Element	Food Matrix	Contaminant/Target Analyte	Detection/Performance	Reference
Nitrocellulose fibers	Conductometric lateral flow	Antibodies	Food samples	*E. coli* O157:H7, Bovine viral diarrhea virus (BVDV)	64 CFU mL^−1^ (bacteria) 103 CCID mL^−1^ (Virus)	Luo et al., 2010 [6]
Cu-based MOF nanofibers (CuMOF)	Amperometric biosensor	Xanthine oxidase (XOD)	Squid, yellow croaker	Hypoxanthine/xanthine	LOD = 0.0023 μM and 0.0064 μM	Wang et al., 2019 [64]
Co-MOF/carbon nanofiber	Amperometric biosensor	Co(TMA)MOF catalyst	Salmon fillet	Xanthine, Uric acid	Xanthine: 96.2 nM; Uric acid: 103.5 nM	Ahmad et al., 2022 [71]
Carbon nanofibers	Chemiresistive biosensor	Salmonella-specific aptamer	Fresh beef	Salmonella	Rapid detection; LOD = 10 CFU mL^−1^ (5 min)	Thiha et al., 2018 [73]
Chitosan-carbon nanofiber + AuNPs	Voltammetric aptasensor	Aptamer	Milk	Salmonella	Rapid detection; 1.23 CFU mL^−1^	Fathi et al., 2021 [74]

**Table 4:** Nanofiber-based resistive gas sensors and E-nose arrays for spoilage VOC detection.

Nanofiber Composition	Sensor Type	Target Gases/Markers	Detection Principle	Application/ Remarks	Detection Performance	Reference
SnO_2_ nanowires decorated with Ag and Pt nanoparticles	Resistive (electronic nose)	H_2_ and NH_3_ mixtures	MOS resistance modulation + metal semiconductor junction effects	Miniaturized array with five temperature-differentiated sensors	(LOD = 0.2–1.2 ppm) rapid response (25 s–5 min), and long-term stability (2 months)	Tonezzer et al., 2022 [81]
3SiO_2_:In_2_O_3_ and SiO_2_:ZnO nanofiber mats	Resistive (E-nose)	Spoilage amines	Resistance variation	Differentiated fish freshness levels	Separating freshness stages over 0–48 h	Andre et al., 2022 [82]
SnO_2_, CuO, In_2_O_3_, ZnO nanofibers	Resistive (E-nose array)	VOCs (e.g., alcohols, amines, aldehydes)	MOS resistance change + PCA pattern recognition	Potential for meat spoilage monitoring	Discrimination of five VOCs (ammonia, ethanol, acetaldehyde, isoprene, and acetone)	Zang et al., 2023 [63]

**Table 5:** Functional roles of electrospun nanofibers as active sensing elements and support platforms.

NF Based Sensing Type	Nanofiber System/Composition	Detection Performance	Function or Benefit	Refs.
Electrospinning used to fabricate the sensor element directly	FITC- and Rhodamine B–labeled chitosan nanofibers	Biogenic amines: 1 ppm	Dual-fluorescence colorimetric sensing of spoilage; visible response for seafood freshness monitoring.	[60]
	PS-based upconversion–plasmonic nanofibers (NaYF_4_:Yb^3+^,Er^3+^ + Au@SiO_2_)	Enhanced fluorescence emission	Hybrid photoresponsive fibers with amplified luminescence for trace contaminant detection.	[54]
	Cu-based MOF nanofibers (XOD–CuMOF)	Hypoxanthine: 0.0023 µMXanthine: 0.0064 µM	Nanofibers act as the active sensing layer for seafood freshness; high electrocatalytic activity and rapid electron transfer.	[64]
Electrospun fibers serving as a support platform for functional sensing components	Au@Ag-functionalized PAN nanofibers (SERS substrate)	Thiabendazole: ≈70 ppb (LOQ)	Hybrid plasmonic nanofiber support producing strong electromagnetic hot spots for ultrasensitive Raman detection.	[55]
	Co-MOF/Carbon nanofibers	Xanthine: 96.2 nM Uric acid: 103.5 nM	Porous conductive CNF scaffold improves analyte adsorption, charge transfer, and catalytic efficiency.	[71]
	Carbon nanowire with Salmonella-specific aptamer	10 CFU mL^−1^, detection in 5 min	Conductive NF network transduces aptamer–pathogen binding events into measurable resistance changes.	[73]
	UiO-66-NH_2_/MWCNT@rGONR aptasensor (on NF support)	Recoveries: 97.8–107.7%, RSD < 5%	MOF–carbon hybrid supported on nanofibers enhances surface area and conductivity for antibiotic (kanamycin) detection.	[77]

**Table 6:** Performance and Practical Assessment of Nanofiber-Based Sensors for Food Quality Monitoring.

Sensor Type	Performance Level	Practical Use Scenario	Cost & Scalability	Environmental Friendliness	Refs.
Colorimetric NF sensors	Moderate sensitivity; qualitative to semi-quantitative	Innovative packaging; low-tech visual monitoring	Very low cost; highly scalable	Contingent upon natural dyes, biodegradable polymers	Sun et al., 2021 [29] Hazarika et al., 2023 [49] Duan et al., 2021 [51] Zhang et al., 2026 [52]
Fluorescent NF sensors	High sensitivity; quantitative	Lab/on-device detection with optics	Medium cost; requires instrumentation	Contingent upon fluorophore stability (potential leaching) and biodegradability of polymers	Quan et al., 2021 [53] Wang et al., 2011 [60] Li et al., 2019 [54]
Electrochemical NF sensors	Excellent sensitivity and selectivity nanomolar–picomolar LOD	Portable food safety devices	Higher fabrication complexity	Contingent upon bioreceptors, additives and polymers. Metal oxides and noble-metal catalysts may increase environmental burden	Wang et al., 2019 [64] Ahmad et al., 2022 [71] Thiha et al., 2018 [73] Fathi et al., 2021 [74]
Resistive gas-sensing NF sensors	Rapid response and IoT capability	Spoilage gas monitoring inside packaging	Moderate; depends on MOS materials	Contingent upon materials. Metal oxides and noble-metal catalysts may increase environmental burden	Tonezzer et al., 2022 [81] Andre et al., 2022 [82] Zang et al., 2023 [63]
